# Shenqi Fuzheng Injection Combined with Chemotherapy for Breast Cancer: A Meta-Analysis of Randomized Controlled Trials

**DOI:** 10.1155/2015/635858

**Published:** 2015-10-01

**Authors:** Yanhong Lv, Guijuan Zhang, Yi Ma, Min Ma, Rui Liao, Jingfang Xiang, Ruixue Chen, Xianxin Yan, Fengjie Bie, Maojie Huang, Shijie Liang

**Affiliations:** ^1^Medical College, Jinan University, 601 Huangpu West Avenue, Guangzhou, Guangdong 510632, China; ^2^The First Affiliated Hospital of Jinan University, 601 Huangpu West Avenue, Guangzhou, Guangdong 510632, China; ^3^Institute of Biomedicine and Department of Cellular Biology, Jinan University, 601 Huangpu West Avenue, Guangzhou, Guangdong 510632, China; ^4^Hangzhou Iron and Steel Company Worker Hospital, 1 Kangjian Road, Gongye Area, Hangzhou, Zhejiang 310022, China

## Abstract

*Purpose.* To evaluate the therapeutic effectiveness and safety of shenqi fuzheng injection (SFI) in the associated chemotherapy of breast cancer.* Methods*. 1247 subjects were included in this study for meta-analysis with RevMan 5.3.* Results*. The clinical curative effective rate (OR = 2.03, 95% Cl [1.44, 2.86], *P* < 0.0001), grades of KPS (OR = 4.11, 95% Cl [2.74, 6.16], *P* < 0.00001), CD3^+^ cells (MD = 7.05, 95% Cl [0.45, 13.64], *P* = 0.04) and CD4^+^ cells (MD = 8.60, 95% Cl [2.67, 14.54], *P* = 0.004) and CD4/CD8^+^ cells (MD = 0.35, 95% Cl [0.14, 0.56], *P* = 0.001), WBC (OR = 0.30, 95% Cl [0.20, 0.46], *P* ≤ 0.0001), PLT (OR = 0.36, 95% Cl [0.20, 0.67], *P* = 0.001), gastrointestinal reaction (OR = 0.21, 95% Cl [0.14, 0.32], *P* < 0.00001), and ECG (OR = 0.26, 95% Cl [0.13, 0.51], *P* < 0.0001) in the experimental group were superior to the control group. While there were no differences between two groups in CD8^+^ (MD = 0.21, 95% Cl [−2.81, 3.23], *P* = 0.89), NK^+^ (MD = 1.06, 95% Cl [−9.40, 11.53], *P* = 0.84), RBC (OR = 0.49, 95% Cl [0.14, 1.74], *P* = 0.27), liver function (OR = 0.59, 95% Cl [0.28, 1.24], *P* = 0.16), renal function (OR = 0.56, 95% Cl [0.13, 2.45], *P* = 0.44), and bone marrow suppression (OR = 0.50, 95% Cl [0.25, 1.01], *P* = 0.05).* Conclusion*. SFI combined with chemotherapy, to some extent, can improve the effectiveness and the security in the treatment of breast cancer; the mechanism may be related to the elevated immunity.

## 1. Introduction

Breast cancer which is duct epithelium abnormal malignant hyperplasia, the most frequently occurring cancer in women, is becoming a major public health problem [1]. In 2012, GLOBOCAN statistics showed that nearly 1.7 million women were diagnosed with breast cancer, with 522,000 related deaths, leading to an increase in breast cancer incidence and related mortality by nearly 18% from 2008 [2]. It has been predicted that the worldwide incidence of female breast cancer will reach approximately 3.2 million new cases per year by 2050 [2]. In addition, 89% of breast cancers in the world were diagnosed from the age of 40 onwards [3]. However, under 40, women who were diagnosed with breast cancer are gradually increasing, especially for the age of 20–40 [4, 5]. Besides, breast cancer patients with lymph node invasion and worsening tumor grade have a poor long-term survival [6]. Therefore, breast cancer has become an utmost important issue with its effect on worldwide health care and economy and the need for urgency for preventive and treatment measures [7].

Currently, the available treatment means for breast cancer included surgery, radiotherapy, chemotherapy, endocrine therapy, and the new biological targeted therapy [8, 9]. However, chemotherapy, in the long term, occupying the dominant position in the nonsurgical treatment for cancer, has made remarkable efficacy in clinical treatment, particularly for reducing tumor size and increasing disease-free recurrence [10]. Unfortunately, the primary drug resistance or acquired drug resistance which heavily obstructed chemotherapy clinical effect is still a major challenge which makes experts, academics, researchers, and doctors puzzled for a long time [11]. Besides, severe toxicities and adverse effects from chemotherapy, such as hematological toxicity, gastrointestinal reaction, and cardiac damage, weaken immunologic system of patients, prolong treatment, and lower survival [12]. At present, more and more researchers devote themselves to the study on how to transform the resistance of chemotherapy and better reduce the adverse effects of chemotherapy, being a huge focus in the chemotherapy research of cancer [13]. New studies [14] have found that the immune regulatory molecules are potentially involved in resistance of chemotherapy, which cause widely concern by scholars and researchers from home and abroad.

In China, a growing number of clinical randomized controlled trials have reported that Chinese medicine herbs especially compound preparations extracted from Chinese natural herbs are beneficial to chemotherapy in enhancing immunity, reducing adverse effects, and decreasing the probability of recurrence and metastasis of advanced cancer [15, 16]. Furthermore, traditional Chinese medicine has gradually been recognized by some foreign areas (Japanese, India, Australia, and Africa), but there is a lack of effective evaluation standard to realize internationalization [17]. In recent years, evidence-based medicine, an available evaluation standard of clinical treatment, supported by entire world has proved that much traditional Chinese medicine is safe and effective and has been accepted by foreign people [18]. Yet few foreign researchers make a systematical evaluation on the effects of Chinese medicine combined with chemotherapy in the clinical treatment.

Currently, shenqi fuzheng injection has been widely used combined with chemotherapy for the treatment of breast cancer in China. So the author uses meta-analysis to conduct a systematic review in terms of the clinical efficacy and safety of SFI combined with chemotherapy in the treatment of breast cancer to clarify whether the combination can really enhance immune function to reverse drug resistance and reduce adverse effects in order to better improve the clinical efficacy.

## 2. Methods

### 2.1. Literature Search Strategy

The PubMed, EMBASE, CENTRAL, China National Knowledge Infrastructure Database (CNKI), Chinese Scientific Journals Full-Text Database (VIP), Wanfang Database, and China Biological Medicine Database (CBM) were searched from these publications established to 12, 2014, with the following keywords: breast cancer and Shenqi fuzheng injection. All the publication languages were restricted to Chinese and English.

### 2.2. Studies Inclusion and Exclusion Criteria

#### 2.2.1. Inclusion Criteria

Included studies must meet the following criteria: ① the disease was diagnosed and confirmed with breast cancer by pathology or imaging studies or the “China common malignant tumor diagnosis and treatment standards” clinical diagnosed criteria or the “Chinese Anti-Cancer Association of breast cancer treatment guidelines and norms” (2011 edition); [19] ② there were randomized controlled trials groups; ③ interventions must be SFI combined with chemotherapy treatment; ④ subjects before being included in the study were not using other anticancer drugs of Chinese herbs; ⑤ there were not heavily damage for liver and kidney function before the subjects included in the study.

#### 2.2.2. Exclusion Criteria

Included studies must meet the following criteria: ① there is no trial randomized control group; ② the language of references was not English or Chinese; ③ nontherapeutic clinical research, animal studies, and review articles; ④ so poor balance between two groups could not be compared; ⑤ research is without relative outcome indicators; ⑥ the latest and most comprehensive data should be extracted from studies with duplicate publication; ⑦ interventions were not the comparison between SFI combined with chemotherapy and chemotherapy alone in the treatment of breast cancer; ⑧ subjects before being included in the study were using other anticancer drugs of Chinese herbs; ⑨ subjects before being included in the study had severe liver and kidney damage.

### 2.3. Documents Screening and Data Extraction

Two researchers (Jingfang Xiang and Ruixue Chen) read the relative studies independently by the title and summary to exclude the references which did not met the inclusion criteria. Then, reading full text in the remaining studies as mentioned above, finally, determines whether these references included were final studies or not, according to the inclusion and exclusion criteria. This course had to be cross-checked in order to ensure accuracy and reliability. All data on patient characteristics, treatment details, and clinical outcomes were independently abstracted by other two investigators (Fengjie Bie and Xianxin Yan) using a standardized data collection form. To avoid subjective bias, the author's name, the title of the paper published in the journal, year, and country must be omitted from data extraction. Disagreements on study inclusion or data extraction were resolved by consensus of three coauthors (Guijuan Zhang, Yi Ma, and Min Ma). The data independently extracted by the remaining investigators (Rui Liao, Shijie Liang, and Maojie Huang) as follows were (1) study design overview, including the study randomization methods, demographic characteristics, and blinding implementation; (2) the sample size of combination group and sample group, the short term clinical efficacy, KPS score, adverse effects, and immune function expression.

### 2.4. Outcome Indicators

Main outcome measures are (1) the treatment efficiency and KPS score improvement; (2) the changes of immune function indexes (CD3^+^, CD4^+^, CD8^+^, CD4/CD8^+^, and NK^+^); (3) adverse effects: the blood toxicity (white cells, red cells, and platelets), gastrointestinal reaction, liver function, renal function, ECG, and bone marrow suppression.

### 2.5. Study Quality Evaluation

According to the Jadad score [20] of randomized controlled trials and the Cochrane evaluation handbook of randomized controlled trials to assess the quality of study [21], the main evaluation contents included randomization, blinding, allocation concealment, follow-up, inclusion/exclusion criteria, and statistical analysis. The score for each article can range from 0 (lowest quality) to 7 (highest quality). Scores of 4–7 represent good to excellent (high quality) and 0 to 3 represent poor or low quality. In addition, the bias parameters of included studies contained random sequence generation (selection bias), allocation concealment (selection bias), the blinding of participants and personnel (performance bias), the blinding of outcome assessment (detection bias), incomplete outcome data (attrition bias), selective reporting (reporting bias), and the other bias. We judged each item on three levels (“Yes” for low bias, “No” for a high risk of bias, and “Unclear”). Then, we assessed the trials and categorized them into three levels: low risk of bias (all the items were categorized “Yes”), high risk of bias (at least one item ranked “No”), and unclear risk of bias (at least one item was “Unclear”).

### 2.6. Statistical Analysis

Meta-analysis was done with Review Manager 5.3 (The Cochrane Collaboration, Oxford, UK). Odds ratio (OR) and 95% confidence intervals (CI) were calculated. Statistical heterogeneity of the results across trials was assessed by Chi-square based* Q*-statistic test, and the inconsistency was calculated by* I* ^2^. If homogeneity (*P* ≥ 0.1,* I* ^2^ ≤ 50%) was not rejected, the fixed-effects model was used to calculate the summary odds ratio (OR) and the 95% CI.

Otherwise, a random-effects model was used when *P* < 0.1,* I* ^2^ > 50%. Also, it was necessary to perform subgroups analysis in order to seek the sources of heterogeneity. Publication bias was evaluated through funnel plots.

### 2.7. Sensitivity Analysis

The total treatment effects in all identified trials related were investigated, and then merged date with summary statistics was extracted from the publication by us.

## 3. Results

### 3.1. Included Trials and Characteristics

**Figure 1:**
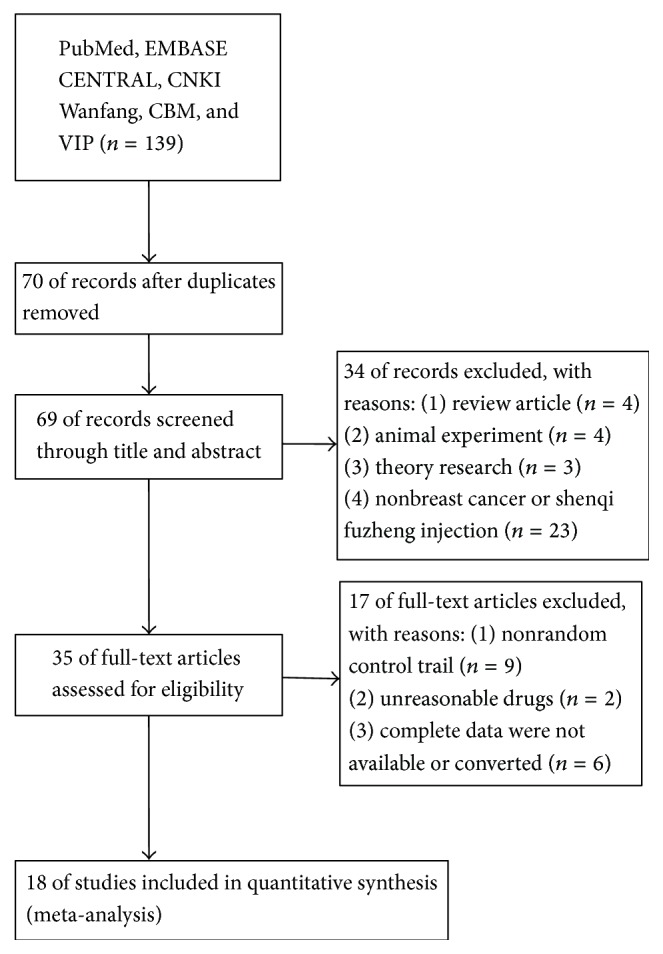
Flow diagram of search and selection of studies.

We firstly retrieved 139 potentially relevant possible studies from electronic database searching. After reading the title, abstract, and full text, excluding the inappropriate studies 121, 18 clinical trials with 1247 breast cancer patients were finally included in this meta-analysis. A flow diagram describing literature search and study selection was shown in Figure 1. The cases of shenqi fuzheng injection combined with chemotherapy and individual chemotherapy were 644 and 603, respectively. The general characteristics of included studies were demonstrated in Table 1.

### 3.2. Evaluation of the Clinical Efficacy

#### 3.2.1. Clinical Curative Efficiency (Figure 2)

In the 18 included trials, 8 trials [22–25, 27, 30, 31, 35] with 596 cases reported clinical curative efficiency. Meta-analysis showed the heterogeneity test (*χ* ^2^ = 3.08, *P* = 0.88,* I* ^2^ = 0%), indicating that there was seldom statistical heterogeneity between studies. Based on the heterogeneity results, a fixed-effects model was applied to calculate the combined OR and 95% CI, which were 2.03 (1.44, 2.86), *P* < 0.0001, indicating that there is a statistically significant difference between groups of SFI combined with chemotherapy and chemotherapy alone, which declares that SFI combined with chemotherapy in the treatment of breast cancer can significantly improve the efficiency of clinical curative effect when compared with chemotherapy alone.

#### 3.2.2. KPS Score Evaluation (Figure 3)

Of 18 trials, 8 trials [25, 27, 30, 33–35, 37, 38], including 545 cases, reported KPS score improvement rates. The result showed that there was no statistical heterogeneity between studies (*χ* ^2^ = 2.51, *P* = 0.93,* I* ^2^ = 0%), indicating that a fixed-effects model was used to calculate the combined OR and 95% CI, which were 4.11 (2.74, 6.16), *P* < 0.00001 indicating that there is a statistically significant difference between two groups, which means that SFI combined with chemotherapy may increase KPS score, further to improve quality of life when compared with chemotherapy alone.

### 3.3. Immune Function (Figure 4)

The mark CD3^+^ of immune function was reported by 5 trials [27, 28, 31, 32, 35], containing 442 patients in the 18 included trials. The result of heterogeneity test (*χ* ^2^ = 125.24, *P* < 0.00001, *I* ^2^ = 97%) in the meta-analysis declared statistically significant heterogeneity between studies. According to this result, the random-effects model was used to calculate the combined mean difference (MD) and 95% CI, which were 7.05 (0.45–13.64), *P* = 0.04, indicating that there exists a statistically significant difference between SFI combined with chemotherapy group and chemotherapy group, which means that SFI combined with chemotherapy in the treatment of breast cancer can increase the levels of CD3^+^ expression.

Six trials [27–29, 31, 34, 35], including 498 patients, reported CD4^+^ expression level. The heterogeneity test showed *χ* ^2^ = 187.25, *P* < 0.00001, and *I* ^2^ = 97% in the meta-analysis, indicating statistically significant heterogeneity between studies. Based on the heterogeneity test, it was appropriate to use random-effects model to calculate the combined MD and 95% CI, which were 8.60 (2.67–14.54), *P* = 0.004, indicating that there is a statistically significant difference between two groups, which explains that SFI combined with chemotherapy in the treatment of breast cancer can significantly improve the CD4^+^ expression level.

Six trails [27–29, 31, 34, 35] with 498 cases that reported CD8^+^ meta-analysis showed that there was statistical heterogeneity between studies in terms of the heterogeneity test (*χ* ^2^ = 62.48, *P* < 0.00001, *I* ^2^ = 92%); therefore, the random-effects model was applied to calculate the combined MD and 95% CI, which were 0.21 (−2.81, 3.23), *P* = 0.89, indicating that there is no statistical difference between two groups, which explains that SFI combined with chemotherapy in the treatment of breast cancer cannot improve the CD8^+^ expression level.

The expression CD4^+^/CD8^+^ was also reported by 6 trials [27–29, 31, 34, 35], which included 498 patients. The heterogeneity test showed *χ* ^2^ = 23.80, *P* = 0.0002, and *I* ^2^ = 79%, indicating large statistical heterogeneity between studies. Based on the heterogeneity test, the random-effects model was used to calculate the combined MD and 95% CI, which were 0.35 (0.14–0.56), *P* = 0.001, indicating that there is a statistically significant difference between two groups, which explains that SFI combined with chemotherapy can significantly improve the expression level of CD4^+^/CD8^+^ in the treatment of breast cancer.

4 trials [28, 31, 34, 35] with 382 cases that reported NK^+^ meta-analysis showed that there was statistical heterogeneity between studies in terms of the heterogeneity test (*χ* ^2^ = 188.75, *P* < 0.00001, and *I* ^2^ = 98%). So the random-effects model was applied to calculate the combined MD and 95% CI, which were 1.06 (−9.40, 11.53), *P* = 0.84, indicating that there is no statistical difference between two groups, which indicates that SFI combined with chemotherapy does not increase the NK^+^ expression level in the treatment of breast cancer.

### 3.4. Safety Evaluation

#### 3.4.1. Safety Evaluation of Blood System (Figure 5)

Of 18 included trials, 8 trials [23–25, 27, 30, 35, 38, 39] including 498 patients reported the decrease of white blood cells (WBC) occurrence rate. Meta-analysis showed the heterogeneity test (*χ* ^2^ = 4.70, *P* = 0.7, *I* ^2^ = 0%), indicating that there was no statistical heterogeneity between studies. Based on the heterogeneity results, a fixed-effects model was applied to calculate the combined OR and 95% CI, which were 0.30 (0.20, 0.46), *P* < 0.00001, indicating that there is a statistically significant difference between two treatment groups, which indicates that SFI combined with chemotherapy can significantly reduce the rate of white blood cells (WBC) decline when compared with chemotherapy alone in the treatment of breast cancer.

The incidence of red blood cells (RBC) decrease was reported by 2 studies [24, 38] with 110 cases. In the meta-analysis, the heterogeneity test showed *χ* ^2^ = 0.09, *P* = 0.77, and *I* ^2^ = 0%, indicating that there was no statistical heterogeneity between studies. Regarding the heterogeneity results, a fixed-effects model was used to calculate the combined OR and 95% CI, which were 0.49 (0.14,0.74), *P* = 0.27, indicating that there is no statistical difference between two treatment groups, which indicates that SFI combined with chemotherapy failed in significantly reducing the rate of hemoglobin decrease, compared with chemotherapy alone, in the treatment of breast cancer.

5 trials [24, 25, 35, 38, 39] containing 337 patients reported the incidence of Platelet (PLT). The heterogeneity test showed *χ* ^2^ = 1.81, *P* = 0.77, and *I* ^2^ = 0%, indicating that there was no statistical heterogeneity between studies. With regard to the heterogeneity results, a fixed-effects model was used to calculate the combined OR and 95% CI, which were 0.36 (0.20, 0.67), *P* = 0.001, indicating that there is a statistically significant difference between two treatment groups, which suggests that SFI combined with chemotherapy can greatly reduce the rate of platelets decline in the treatment of breast cancer when compared with chemotherapy alone.

#### 3.4.2. Nonhematologic Safety Evaluation (Figure 6)

The change of liver function was reported by 3 trials [24, 25, 35] with 219 patients from 18 included studies. The result (*χ* ^2^ = 0.26, *P* = 0.88, and *I* ^2^ = 0%) was showed by the heterogeneity test in the meta-analysis. Regarding this conclusion, the fixed-effects model was applied to calculate the combined OR and 95% CI, which were 0.59 (0.28, 1.24), *P* = 0.16, indicating that there is a statistical difference between two treatment groups, which explains that SFI combined with chemotherapy in the treatment of breast cancer can reduce the incidence of liver function injury when compared with chemotherapy alone.

2 trials [25, 35] which included 155 cases reported the incidence of renal function changes; the heterogeneity test showed *χ* ^2^ = 0.13, *P* = 0.72, and *I* ^2^ = 0%, indicating that the statistical heterogeneity existed in the studies. Based on the heterogeneity results, a fixed-effects model was applied to calculate the combined OR and 95% CI, which were 0.56 (0.13, 2.45), *P* = 0.44, indicating that there is no statistical difference between two treatment groups, which suggests that SFI combined with chemotherapy in the treatment of breast cancer fails to reduce the damaging incidence of renal function when compared with chemotherapy alone.

Of 18 studies included in the trials, 7 studies [22–24, 30, 32, 35, 39] with 470 cases reported gastrointestinal adverse effects (nausea or vomiting) incidence; the heterogeneity test showed *χ* ^2^ = 3.7, *P* = 0.72, and *I* ^2^ = 0%, indicating that there was no statistical heterogeneity between studies. Based on the heterogeneity results, a fixed-effects model was applied to calculate the combined OR and 95% CI, which were 0.21 (0.14, 0.32), *P* < 0.00001, indicating that there is a statistically significant difference between two treatment groups, which suggests that SFI combined with chemotherapy in the treatment of breast cancer can greatly reduce the incidence of gastrointestinal adverse reactions when compared with chemotherapy alone.

The incidence of ECG change was reported by 5 trials [22, 26, 35, 36, 39] with 331 patients. The result (*χ* ^2^ = 0.85, *P* = 0.93, and *I* ^2^ = 0%) was showed in the heterogeneity test. Regarding this conclusion, a fixed-effects model was applied to calculate the combined OR and 95% CI, which were 0.26 (0.13, 0.51), *P* < 0.0001, indicating that there is a statistically significant difference between two treatment groups, which explains that SFI combined with chemotherapy can reduce the incidence of cardiac damage when compared with chemotherapy alone in the treatment of breast cancer.

2 trials [22, 24] including 154 patients reported the incidence of bone marrow suppression change. The heterogeneity test showed *χ* ^2^ = 0.00, *P* = 0.98, and *I* ^2^ = 0%, indicating that there was statistical heterogeneity between studies. With regard to the heterogeneity results, a fixed-effects model was used to calculate the combined OR and 95% CI, which were 0.50 (0.25–1.01), *P* = 0.05, indicating that there is a statistical difference between two treatment groups, which indicates that SFI combined with chemotherapy compared with chemotherapy alone can reduce the damaging incidence of bone marrow suppression in the treatment of breast cancer.

### 3.5. Risk of Bias of Studies

18 trials included in meta-analysis were reported as RCTs; only 8 trials described clearly the methods of grouping, indicating that there has been a possibility of high selectivity bias in our study. It was not clear that grouping was concealment; whether the results of the research object, the implementers of plan, and the measurer of effect in 18 trails were used blind method to study, it did not describe, implying a possibility of high implementation bias have existed in our study. 1 article was reported with cases of follow-up. As for study baseline, 18 trials described baseline information in detail about research object, such as gender and age. According to the Jadad scale (the detailed contents were presented in Table 2), 14 studies were of low quality, with a quality score of 3, and only 4 studies were of moderate quality, with a quality score of 4. Characteristics and quality of all included studies are presented in Figures 9 and 10.

### 3.6. Publication Bias Analysis

**Figure 7:**
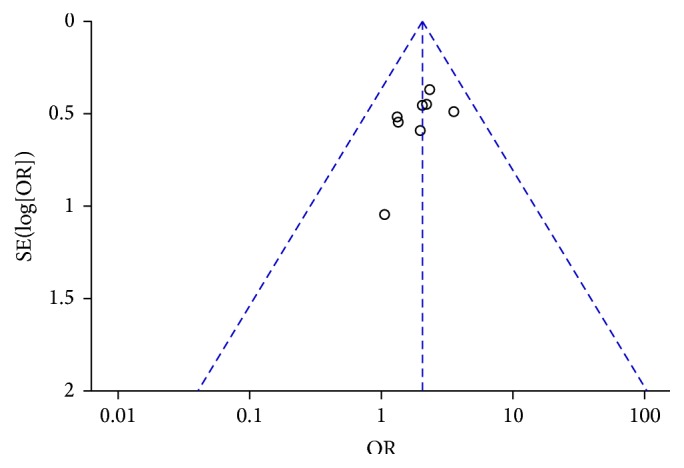
Funnel plot of clinical curative efficiency.

**Figure 8:**
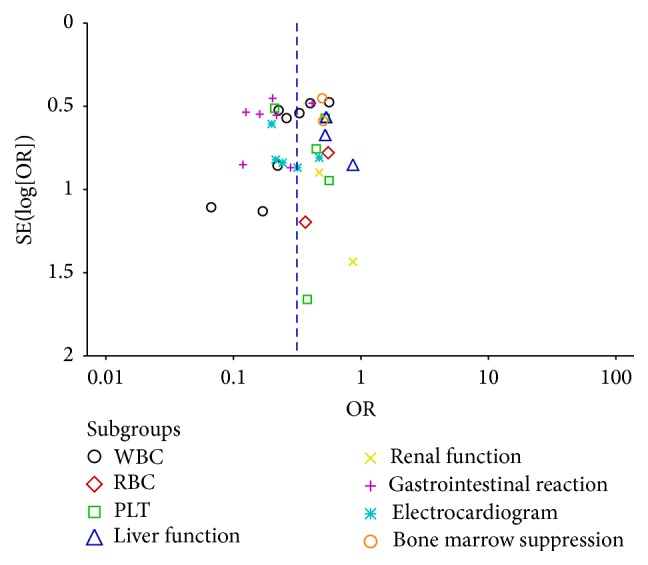
Funnel plot of safety.

**Figure 9:**
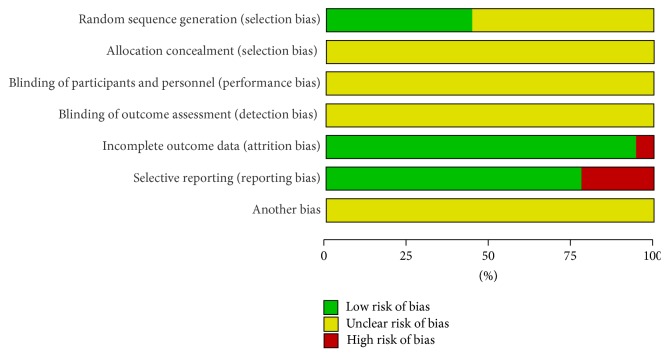
Risk of bias graph: review authors' judgments about each risk of bias item presented as percentages for all included studies.

**Figure 10:**
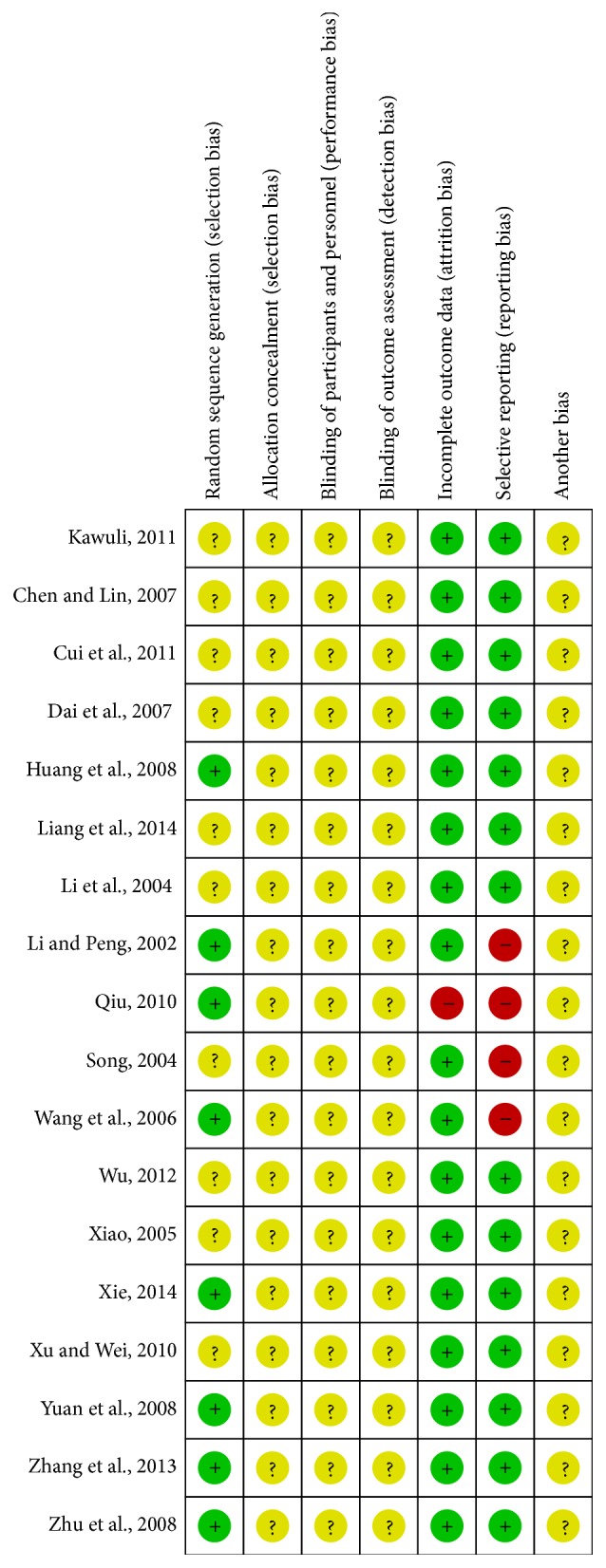
Risk of bias summary: review authors' judgments about each risk of bias item for each included study.

Figures 7 and 8 are the funnel plot based on studies with data on clinical efficacy and safety. Results showed that all points in the funnel plots were asymmetrical, indicating that publication bias may have existed in our study which might influence the results of our analysis.

## 4. Discussion

At present, most of the anticancer drugs used in chemotherapy have cytotoxic injury to normal cells and further induce immunodepression, seriously affecting patients' quality of life, ignoring their great curative effects, and even leading to significant morbidity and mortality, which is a major limiting factor in clinical chemotherapy without efficacious remedies [40, 41]. A large number of clinical trials have proved that tradition Chinese medicine can really repair and improve the cancer patients' immunity, directly against chemotherapy-induced immunosuppression, help patients smoothly cross the chemotherapy, and further prolong survival [42, 43].

Huáng qí and dang shen are most widely used traditional Chinese herbal medicines for improving the immunity of patients [43]. According to the records of* Compendium of Materia Medica* that written by Li Shizhen, which is a famous and classical work of Chinese traditional medicine, dang shen has an effect of tonifying internal organs and qi and nourishing spleen and lung power, while huangqi can tonify qi and strengthen exterior. Making the combination of the two herbs, can greatly enhance the function of Fuzheng Guben and tonifying Qi. In other words, they can rapidly improve the ability of body against disease. Now, shenqi fuzheng is a newly developed injection concocted from traditional Chinese medicinal herbs: Radix Astragali (huáng qí) and Radix Codonopsis (dang shen), with a rate of 1 : 1, approved by the State Food and Drug Administration of the People's Republic of China in 1999 primarily as an antitumor injection to be manufactured and marketed in China [44, 45].

This review suggested that SFI intervention indeed improves the clinical effect and the quality of survival (KPS) and strengthens immune function (CD3^+^, CD4^+^, and CD4^+^/CD8^+^), meanwhile reducing the adverse of chemotherapy such as blood toxicity (WBC, PLT), gastrointestinal reaction, heart injury, and bone marrow suppression. But it cannot play an important part in CD8^+^, NK, RBC, liver function, and renal function, considering that it may be closely related with the small sample size included. If there are large trials samples, in the future, to be further researched, believing SFI intervention may take effect in the above aspects.

Modern pharmacological study found that the effective components of Radix Codonopsis included sterol, triterpenes, glycoside, alkaloid, and polysaccharide. It has the functions of antitumor and improving cellular immunity by modulating macrophage-mediated immune responses [46]. The major components of Radix Astragali are astragalosides and the other pharmacological ingredients include polysaccharides, flavones, and amino acids. It also plays an important role in antitumor by significantly blocking the production of tumor necrosis factor (TNF-*α*) and generating interleukin-2 to enhance immunity [47]. Therefore, SFI can activate immune system and inhibit tumor growth. Currently, the meta-analysis provided evidence on the effectiveness of clinical treatment which can not only help to solve a major public health problem that would benefit patients directly, but also can be used as a reliable evidence to guide clinical practice and make a reasonable health policy. That is why it obtained the unprecedented attention in the field of worldwide medicine [48]. Therefore, this paper made the effectiveness and safety system evaluation of shenqi fuzheng injection combined with chemotherapy for the treatment of breast cancer by meta-analysis, aiming at providing the scientific basis for worldwide medicine in this field.

However, this systematic review also has limitations and shortcomings. Firstly, the literature included was published in China, which may form language bias and publication bias, leading to the emergence of inconstant result. Secondly, in 18 included trials, only 8 trials mentioned detailed random allocation method; allocation concealment and blinding were not described in all included trials, which may result in the emergence of high selectivity bias and performance bias, leading to overestimating the efficacy of the treatment group. Thirdly, only 1 trial reported follow-up, unable to judge the long-term efficacy, so there may be a possibility of selective reporting bias. In all, the evidence from this study may be insufficient and need to be further confirmed.

## 5. Conclusion

Shenqi fuzheng injection combined with chemotherapy in the treatment of breast cancer may really enhance the immunity of patients to improve the clinical efficacy and safety. But the detailed mechanism of how shenqi fuzheng injection works in chemotherapy is not absolutely clear so far and the quality of included studies were relatively inadequate. Hence, it is necessary to carry out more high quality, large sample, multicenter, prospective, randomized, double blind clinical trials to be further confirmed in the future.

## Figures and Tables

**Figure 2:**
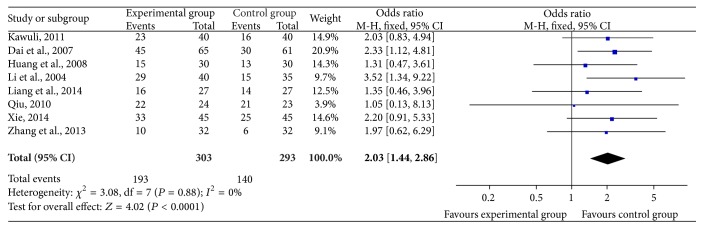
Forest plot of improved clinical curative efficiency.

**Figure 3:**
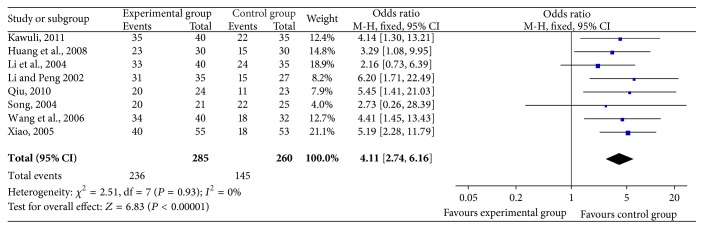
Forest plot of improved KPS.

**Figure 4:**
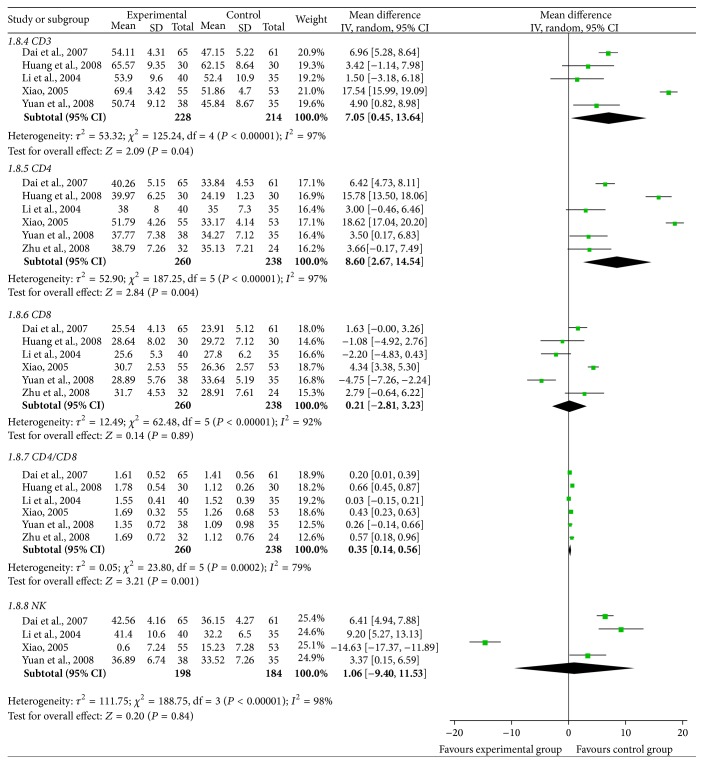
Forest plot of immune function.

**Figure 5:**
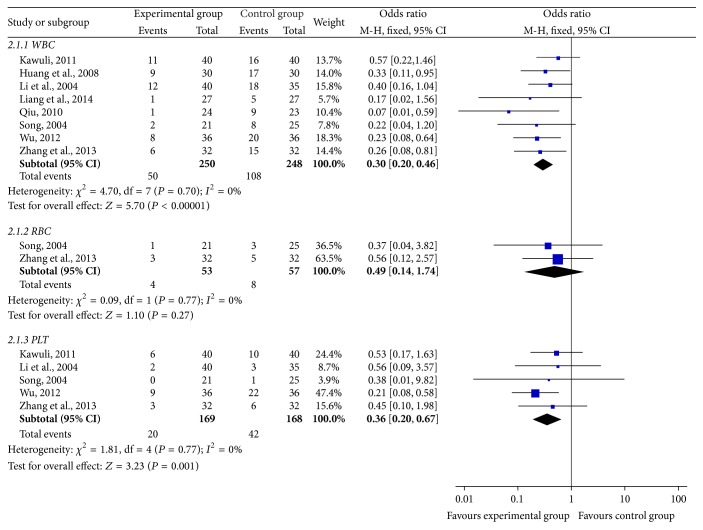
Forest plot of blood system.

**Figure 6:**
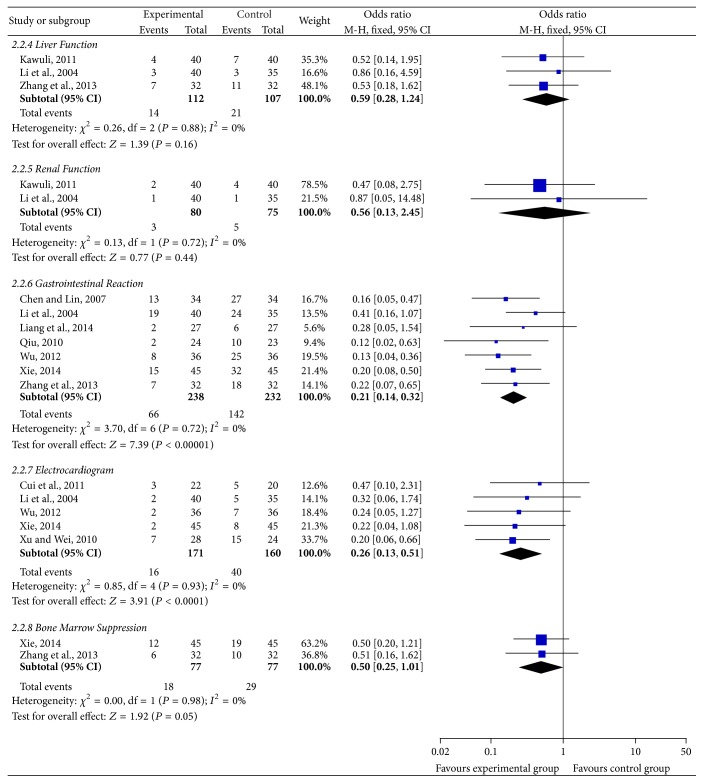
Forest plot of nonhematologic system.

**Table 1:** Study characteristics and quality.

First author, year, country	Sample size (E/C)	Age (F)	TNM	Intervention (E/C)	Treatment course (C/W/D)	KPS	Jadad score
Xie, 2014, [22] China	45/45	35~68	—	SFI + CAF	4~6 C, 3 W/C	—	4
Liang, 2014, [23] China	27/27	29~57	III-IV	SFI + CTF	2 C, 21 D/C	≥60	3
Zhang, 2013, [24] China	32/32	32–67	—	SFI + GEM, CDDP	21 D	≥60	4
Kawuli, 2011, [25] China	40/40	28~65	III-IV	SFI + TPX, E-ADM	3 C, 3 W/C, 7 D/W	≥60	3
Xu, 2010, [26] China	28/24	47/49	—	SFI + TA	4 C, 21 D/C	≥60	3
Huang, 2008, [27] China	30/30	47/46	III-IV	SFI + CTF	2 C, 21 D/C	≥60	4
Yuan, 2008, [28] China	38/35	19~60	II-III	SFI + CAF	20 D	>60	3
Zhu, 2008, [29] China	32/24	52.5/51	I–III	SFI + CEF	10 D	—	4
Qiu, 2010, [30] China	24/23	52.04/52.17	III-IV	SFI + TPX, E-ADM	2 C, 3 W/C	≥60	3
Dai, 2007, [31] China	65/61	26~70	II-III	SFI + CEF	2 C, 28 D/C	>80	3
Chen, 2007, [32] China	34/34	38~64	I-II	SFI + CEF	6 C, 21 D/C	—	3
Wang, 2006, [33] China	40/32	45.2 ± 9.8/46.7 ± 0.5	—	SFI + 5-FU, E-ADM, CTX	6 C, 21 D/C	≥60	3
Xiao, 2005, [34] China	55/53	43~63	—	SFI + FEC	8 D	≥60	3
Li, 2004, [35] China	40/35	56.4/54.2	IV	SFI + NE	3 C, 28 D/C	≥80	3
Cui, 2011, [36] China	22/20	33~62	—	SFI + FAC, AC	4–6 C, 5–8 D/C	>80	3
Li, 2002, [37] China	35/27	47.2 ± 10.8/46.7 ± 10.5	—	SFI + 5-FU, CTX, MMC	3 C, 21 D/C	>50	3
Song, 2004, [38] China	21/25	52/58	II-III	SFI + CMF	2 C, 2 W/C	—	3
Wu, 2012, [39] China	36/36	35~69	—	SFI + CMF	4 C, 28 D/C	—	3

Note: E/C: experimental group/control group; F: female; TNM: T: tumor, N: lymph node, and M: metastasis; C: cycle; W: week; D: day; KPS: Karnofsky; SFI: shenqi fuzheng injection; CAF: CTX (cyclophosphamide) and ADM (Adriamycin) and 5-FU (5-fluorouracil); CTF: CTX and THP (Therarubicin) and 5-FU; TA: PTX (Paclitaxel) and E-ADM/EPI (epirubicin); CEF: CTX and E-ADM/EPI and 5-FU; TE: PTX and EPI; FEC: CTX and 5-FU and EPI; NE: NVB (Vinorelbine) and E-ADM; FAC: 5-FU and ADM and CTX; AC: ADM and CTX; CMF: CTX and MTX (Methotrexate) and 5-FU; CDDP: cisplatin; MMC: mitomycin; GEM: gemcitabine; Jadad score: modified Jadad scale that was used.

**Table 2:** The modified Jadad scale.

Item	Score
Randomization	
Not randomized or inappropriate method of randomization.	0
The study was described as randomized and the method of randomization was appropriate.	2
Concealment of allocation	
Not describing the method of allocation concealment.	0
The study was described as using allocation concealment method and it was appropriate.	1
Double blinding	
No blind or inappropriate method of blinding.	0
The study was described as double blind and the method of it was appropriate.	1
Withdrawals and dropouts	
Not describing the follow-up.	0
A description of withdrawals and dropouts.	1
Inclusion/exclusion criteria	
No clear description of the inclusion/exclusion criteria.	0
A clear description of the inclusion/exclusion criteria.	1
Statistical analysis	
Not describing the method of statistical analysis.	0
Describing the method of statistical analysis.	1
